# Exploring the evolutionary journey of the lumpy skin disease virus through the phylogenetic and phylo-geo network analysis

**DOI:** 10.3389/fcimb.2025.1575538

**Published:** 2025-06-04

**Authors:** Manjunatha Reddy Gundallahalli Bayyappa, Manoj Kumar Goud Pyatla, Sai Mounica Pabbineedi, Narasimha Tanuj Gunturu, Sai Manohar Peela, Sudeep Nagaraj, Sunil Tadakod, Ravi Kumar Gandham, Baldev Raj Gulati

**Affiliations:** ^1^ Capripoxvirus Lab, Veterinary Pathology, Indian Council of Agricultural Research (ICAR)-National Institute of Veterinary Epidemiology and Disease Informatics (NIVEDI), Bengaluru, Karnataka, India; ^2^ Animal Biotechnology Division, Indian Council of Agricultural Research (ICAR)-National Bureau of Animal Genetic Resources, Karnal, Haryana, India

**Keywords:** lumpy skin disease virus, TMRCA, phylogenetic analysis, haplotype network, genetic diversity, transboundary spread

## Abstract

**Introduction:**

Lumpy Skin Disease Virus (LSDV), an emerging pathogen from the *Capripoxvirus* genus, continues to challenge global livestock health with its expanding host range and genetic adaptability.

**Materials and methods:**

In this study, we report the first isolation and whole genome sequencing of LSDV from Bos frontalis, a semi-domesticated bovine species native to Northeast India, along with the assembly of an isolate from cattle.

**Results:**

Time to the Most Recent Common Ancestor (TMRCA) estimates support a relatively recent common origin for Indian strains, pointing to ongoing virus circulation and regional adaptation. The maximum likelihood phylogenetic tree of the whole genome and G protein-coupled chemokine receptor (GPCR) gene further demonstrated the clustering of global strains, emphasizing the virus’s transboundary movement and genomic diversity. To strengthen phylogenetic inference, we identified shared SNPs, synonymous and non-synonymous mutations across the genome with a total of 2212 variants. Haplotype network and mutation pattern analyses across global genomes further highlighted the conservative evolution of Indian isolates within a distinct haplogroup.

**Discussion:**

Several mutation events between haplogroups highlight the virus’s continuous genetic diversification, which correlates with known patterns of spread.

## 1 Introduction

In recent years, (1)there has been a notable rise in significant transboundary emerging diseases affecting various animal species, posing substantial threats to both economic stability and public health, especially in relation to food security (Bianchini et al., 2023). One such disease that has garnered attention is lumpy skin disease (LSD), which has led to substantial economic losses within the cattle industry. Recognizing its swift transmission and reemergence, the World Organisation for Animal Health (WOAH) has classified LSD as a critical notifiable disease (Tuppurainen et al., 2017). The causative agent, the lumpy skin disease virus (LSDV), belongs to the Poxviridae family, within the Capripoxvirus genus, and is classified alongside the sheep pox virus (SPPV) and goat pox virus (GTPV) (Al-Salihi, 2014).

LSD spreads primarily through arthropod vectors such as biting flies, mosquitoes, and ticks. This makes the disease more prevalent epidemiologically during the summer when hot and humid conditions favor vector activity. Rarely, the direct transmission can occur through close contact between infected and susceptible animals and contaminated feed and water sources (Al-Salihi, 2014; Reddy et al., 2023). The disease leads to high rates of morbidity with varying levels of mortality, and clinically, affected animals exhibit symptoms such as fever, reduced appetite, swollen lymph nodes, and characteristic skin lesions distributed across the body. In severe cases, animals experience diminished production ability, infertility, decreased milk production, and compromised hide quality (Babiuk et al., 2008b; Manjunatha Reddy et al., 2025).

The history of LSD outbreaks traces back to 1929 in what is now Zambia, formerly North Rhodesia, located in South Africa (Bianchini et al., 2023). Until the 1980s, occurrences of LSD were sporadic and primarily confined to the African subcontinent. However, outbreaks beyond Africa emerged in Egypt in 1988, followed by Israel in 1989, and subsequently in several Middle Eastern countries (Sprygin et al., 2019). The disease then spread to European nations, with Turkey experiencing outbreaks in 2013, followed by the Balkans and Russia in 2015 (Mazloum et al., 2023). In 2019, the disease reached the Asian subcontinent, with reports from China, Bangladesh, India, Vietnam, Thailand, Mongolia, Pakistan, Sri Lanka, Myanmar, and Afghanistan (Giasuddin et al., 2019; Sudhakar et al., 2020; Lu et al., 2021). Genetic analysis of LSD isolates from its emergence until 2015 suggested a common origin. However, in 2017, the first recombinant strain was reported in Saratov, Russia, followed by reports from Udmurtya, Russia, in 2018 and Xinjiang, China, in 2019 (Ma et al., 2022). These recombinant strains subsequently spread to other nations such as Mongolia, Vietnam, and Thailand (Sprygin et al., 2022; Mathijs et al., 2021; Seerintra et al., 2022). Notably, in 2019, LSD outbreaks in southeastern parts of the Asian subcontinent, including Pakistan, Bangladesh, India, Myanmar, Sri Lanka, and Pakistan, were attributed to the KSGPO-like vaccine strain (Mazloum et al., 2023; Manjunathareddy et al., 2024).

LSDV, belonging to the large DNA virus family Poxviridae, shares the characteristic large linear double-stranded DNA, measuring 151 kbp and containing 156 putative open reading frames (ORFs). Poxviruses, including LSDV, are known for their slow evolutionary rate, with genome variations primarily occurring in the flanking regions and in genes related to immune evasion, while most of the genome remains highly conserved (Breman et al., 2023). Given LSDV’s rapid global spread, understanding its evolutionary trajectory is crucial. Since the initial complete genome sequence of LSDV was reported in 2001 (Tulman et al., 2001), numerous sequences have been added to GenBank, providing valuable insights into its evolutionary dynamics. However, genomic data and evolutionary studies on LSDV in India remain limited. Therefore, to elucidate the evolutionary status and molecular epidemiology of LSDV in India, this study determined and analyzed the complete genome sequence of LSDV isolated from cattle in the country.

## 2 Materials and methods

### 2.1 Ethics statement

The study involved the collection of biological samples from cattle. Skin scabs were collected following standard protocols without using anesthesia. Permission for sample collection was granted by the respective states’ Animal Husbandry Departments, and animal owners’ consent was obtained before sampling.

### 2.2 Study area and clinical samples

The samples were collected from various districts of Andhra Pradesh, Arunachal Pradesh, Gujarat, Karnataka, Madhya Pradesh, Maharashtra and Nagaland and during the 2020–2022 outbreaks (*n*=15). Skin scabs were collected from the affected animals and transported in the viral transport medium (VTM) to the National Institute of Veterinary Epidemiology and Disease Informatics (NIVEDI), Bengaluru, India, for subsequent analysis. The scab tissue samples were triturated and 10% suspension was prepared in phosphatebuffered saline (PBS, pH 7.2), followed by filtration using a 0.45 µM syringe filter and stored at -80°C until further use.

### 2.3 Molecular identification and virus isolation

The DNA extraction from the processed samples was done using the DNeasy Blood and Tissue Kit (Catalogue no. 69506, Qiagen, Germany) following the manufacturer’s guidelines. The extracted DNA was subjected to the Capripoxvirus-specific PCR targeting the major enveloped protein (P32) gene (237 bp) (Reddy et al., 2015). Further, the full-length G protein-coupled chemokine receptor (GPCR) gene was amplified as described earlier (Manjunatha Reddy et al., 2023). After confirmation, the processed tissue samples were used for virus isolation. For this, Madin-Darby Bovine Kidney cells (MDBK) maintained in 10% growth media (MEM with 10% FBS) were grown to 90% confluency in 25 cm^2^ flasks and inoculated with the tissue sample suspension. The infected flasks were incubated at 37°C in a 5% CO2 incubator and observed daily for the presence of cytopathic effects (CPE).

### 2.4 GPCR full-length gene sequencing and phylogenetic analysis

The purified PCR products of the GPCR gene were sequenced using their respective forward and reverse primers using the Sangers sequencing method at Eurofins Genomics India Private Limited, Bangalore, India. Further, the sequences were analyzed and edited using the Gene tool (Informer Technologies, Inc.). The nucleotide sequences of the *GPCR* gene of the genus Capripoxvirus, both from Indian origin and other countries, were taken from the GenBank database to conduct phylogenetic analysis. Sequence alignment was done by using the Multiple Alignment using Fast Fourier Transform (MAFFT) tool (MAFFT alignment and NJ/UPGMA phylogeny (cbrc.jp)). Model selection and phylogenetic analysis were constructed by using the IQ-Tree web server with 1000 bootstrap values (Trifinopoulos et al., 2016).

### 2.5 Whole genome sequencing

For the whole genome sequencing one each from first wave (2020-21) of LSD (LSDV/CHITRA-05/NIVEDI/ICAR/2020), second wave (2022-23; ICAR/NIVEDI/LSDV/Mithun/Arunachal Pradesh/2023/India) and third wave (2023-24; ICAR/NIVEDI/LSDV/Cattle/2024/Telegana/India) from two different species cattle, mithun and cattle, respectively were subjected to whole genome sequencing. Briefly, the virus was bulk-produced and concentrated by the polyethylene glycol (PEG) precipitation method. Further, the viral DNA was extracted by using a DNeasy Blood and Tissue Kit (Catalogue no. 69506, Qiagen, Germany) as per the manufacturer’s instructions. The concentration of the DNA was assessed using a Nano spectrophotometer (Nabi), and the DNA was sent to Eurofins Genomics Private Limited, Bangalore, India for sequencing the whole genome (Illumina platform).

### 2.6 Whole genome assembly and annotation

DNA libraries were prepared with an insert size of 150 for Illumina sequencing and sequenced on the Illumina platform. The quality of raw Illumina reads was assessed by FastQC (Andrews, 2010), following which the trimming and filtering were done by Trimmomatic v0.38 (Bolger et al., 2014). The genome was assembled using a *de novo* approach implemented in SPAdes v3.14.0 (Bankevich et al., 2012). LSDV-Neethling strain genome (Accession No: NC_003027) was used as a reference for Reference Assisted Genome Ordering Utility (RAGOUT) (Kolmogorov et al., 2014) assisted assembly of contigs generated by SPAdes assembler. Gaps in the consensus sequence were filled by SOAPdenovo2-GapCloser (Luo et al., 2012) using Illumina paired-end reads. Genome assembly quality was assessed by Quast v.5.2.0 (Gurevich et al., 2013). Annotation was performed with Genome Annotation Transfer Utility (GATU) (Tcherepanov et al., 2006). After the annotation, the whole genome sequence was submitted to the GenBank with Accession number OR863389, PQ510118 and OR602866. The final assembly was mapped against Illumina paired-end reads and the resulting bam file was used to calculate nucleotide wise depth using SAMtools (Li et al., 2009).

### 2.7 Nucleotide sequence retrieval

**Figure 1:**
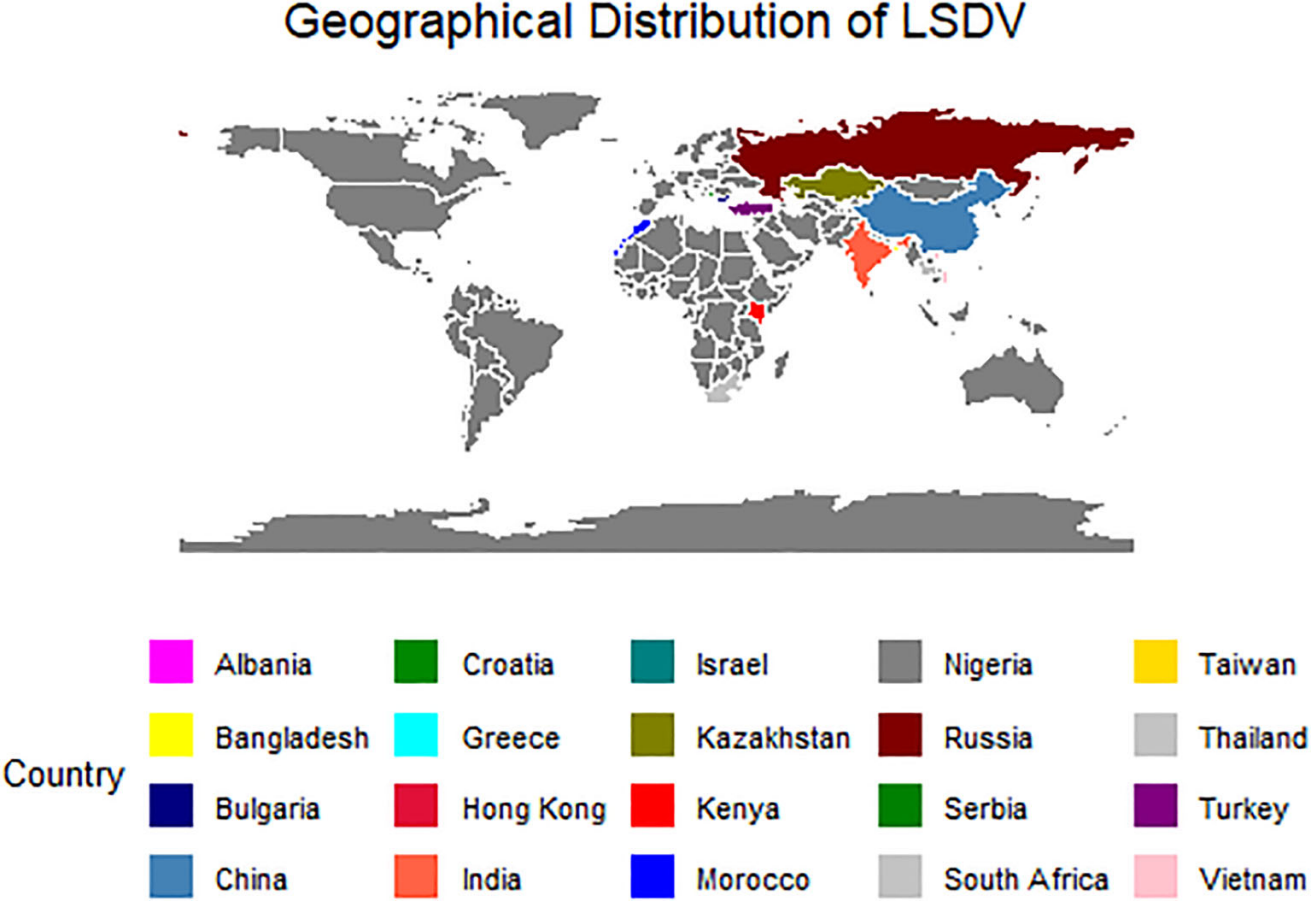
The map depicting the geographical distribution of the LSDV sequences retrieved from PubMed for the present study.

The complete genome sequence of LSDV (~150Kbp) of 113 isolates from worldwide was retrieved from the National Centre for Biotechnology Information (NCBI) Virus database (NCBI Virus (nih.gov)). The geographical distribution of these LSDV sequences, is shown in **Figure 1**. The reference sequence of GTPV (NC004003) and SPPV (NC004002) were downloaded from the NCBI Virus database to investigate the outgroup between all three virus groups.

### 2.8 Phylogenetic analysis of LSDV isolates

The final dataset consisting of 116 sequences was used for phylogenetic analysis. The multiple sequence alignment was performed using the online MAFFT tool (MAFFT alignment and NJ/UPGMA phylogeny (cbrc.jp)). Model selection was determined by IQ Tree and TVM+F+G4 was found to be best fitting model and phylogenetic analysis was carried out by using the IQ-Tree web server construction using the maximum likelihood nucleotide model (Trifinopoulos et al., 2016). Bootstrap resampling with 1,000 replicates was used to test the reliability of the phylograms. The output phylogenetic trees generated were then exported to iTOL (iTOL: Interactive Tree Of Life (embl.de)) an online software tool to visualize, construct and modify phylogenetic trees (Letunic and Bork, 2024). Simultaneously, JModel Test 2.0 was employed to determine the suitable best-fit evolutionary model (Darriba et al., 2012) and Bayesian phylogenetic analysis was done by using Bayesian Evolutionary Analysis by Sampling Trees (BEAST) 2.0 software. For this, nine sequences were excluded due to their similarity with other sequences. Bayesian evolutionary analysis utility software was employed to generate the XML input file for BEAST analysis with the GTR+G model of nucleotide substitution, a constant population size coalescent prior, and strict and relaxed clock models. The sequences were tip-dated according to the year of collection. The best model is selected by calculating the Bayes factor (BF). The phylogenetic analysis was conducted by BEAST 2.7.6 (Bouckaert et al., 2019) with Beagle v4.0.0. library program (Suchard and Rambaut, 2009) to generate and run a Bayesian inference of phylogeny with the Markov chain Monte Carlo (MCMC) (Drummond et al., 2002) algorithm with a chain length of 60 million iterations. MCMC chain convergence was assessed by evaluating the estimated sampling size by using the Tracer v1.7.2 (Rambaut et al., 2018). The clock rate and the Time to the Most Recent Common Ancestor (TMRCA) estimates were extracted by using Tracer v1.7.2 and the final Maximum Clade Credibility tree (MCC) using a posterior was identified by using TreeAnnotator v2.7.6 with a burnin of 10% discarding the first 10% of the trees. The output generated was visualized in FigTree (ed.ac.uk) to construct and modify the tree.

### 2.9 Genomic variant analysis and ORF-level annotation in LSDV

The LSDV sequences were mapped against reference sequence LSDV NI-2490 (NC003027) using Minimap2 (Li, 2021). The sequence alignment and map (SAM) file was then converted and sorted to a binary alignment map (BAM) file using SAMtools (Li et al., 2009). The variants were called from these BAM files using BCFtools (Danecek et al., 2021). The variant call format (VCF) was then annotated using SnpEff, in which a custom database was built for LSDV using Neethling strain genome (Accession No: NC003027) (Cingolani et al., 2012). The isolates were then grouped into 5 based on the clustering pattern observed in phylogenetic analysis. The variants shared in common within the groups and the unique variants within the groups and their position in the ORFs and their effect were evaluated.

### 2.10 Haplotype network and statistical analysis

The multiple sequence alignment file was used to make a Transitive Consistency Score network (TCS network) using PopARTv1.7 (Population Analysis with Reticulate Trees) (Clement et al., 2002; Leigh and Bryant, 2015). Using PopART, the population genetics among the sequences were analyzed, and Tajima’s D value is calculated. The total number of segregating sites, nucleotide diversity, and the number of parsimony information sites were obtained using PopARTv1.7. The sequences used in the study are given in the **Supplementary File**.

## 3 Results

### 3.1 Molecular identification and virus isolation

**Figure 2:**
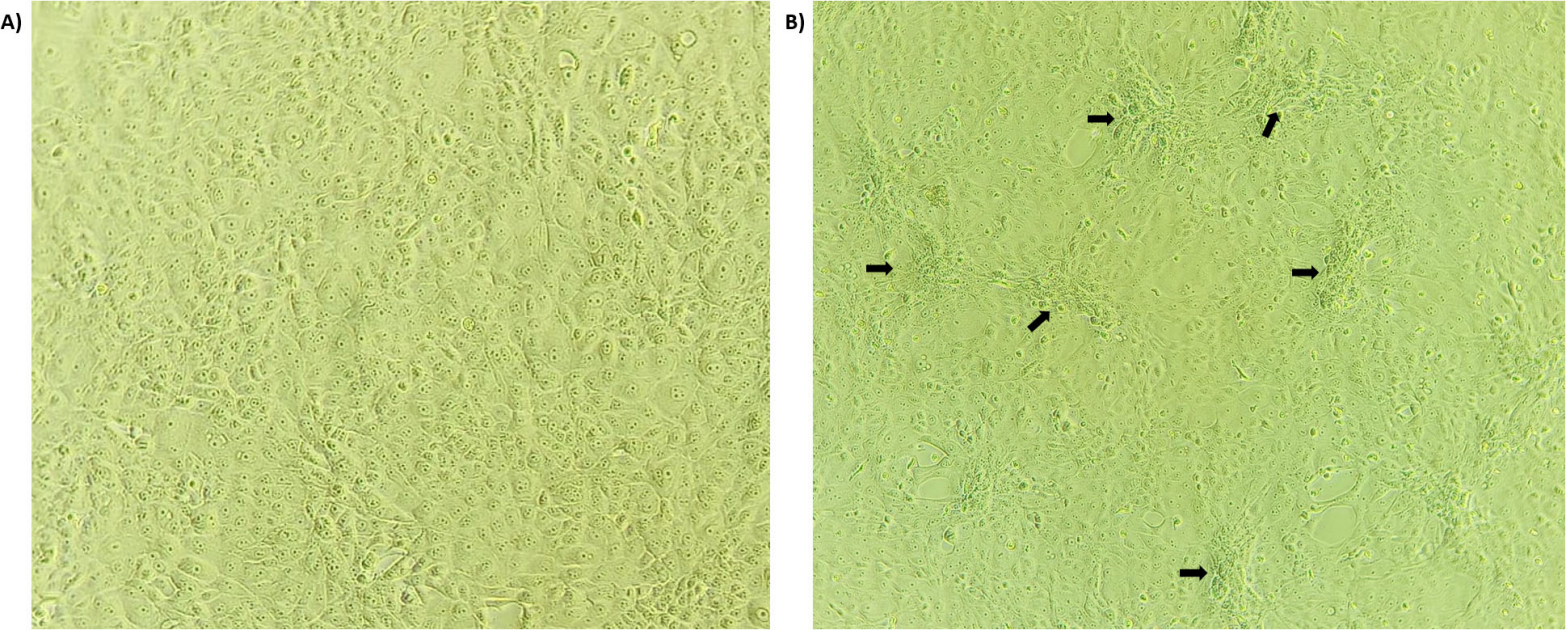
LSD virus isolation: The LSDV (5-Chitra isolate) infected MDBK cell line at 72hrs post-infection. The arrow indicates the clustering, rounding and characteristic foci formation of the MDBK cells infected by LSDV **(B)** compared to healthy cells **(A)**.

DNA extracted from all scab tissue samples collected from different states amplified for the Capripoxvirus-specific gene target P32 of 237 bp, confirming that the virus is a Capripoxvirus. For virus isolation, the MDBK cells were infected with the isolate and observed for 7 days post-infection for any changes in the cell morphology. All samples exhibited morphological changes after 4–5 blind passages. Characteristic CPE, including clustering, rounding of cells, and foci formation, were observed 72–96 hours post-infection (**Figures 2A, B**).

### 3.2 GPCR sequence alignment and phylogenetic analysis

**Figure 3:**
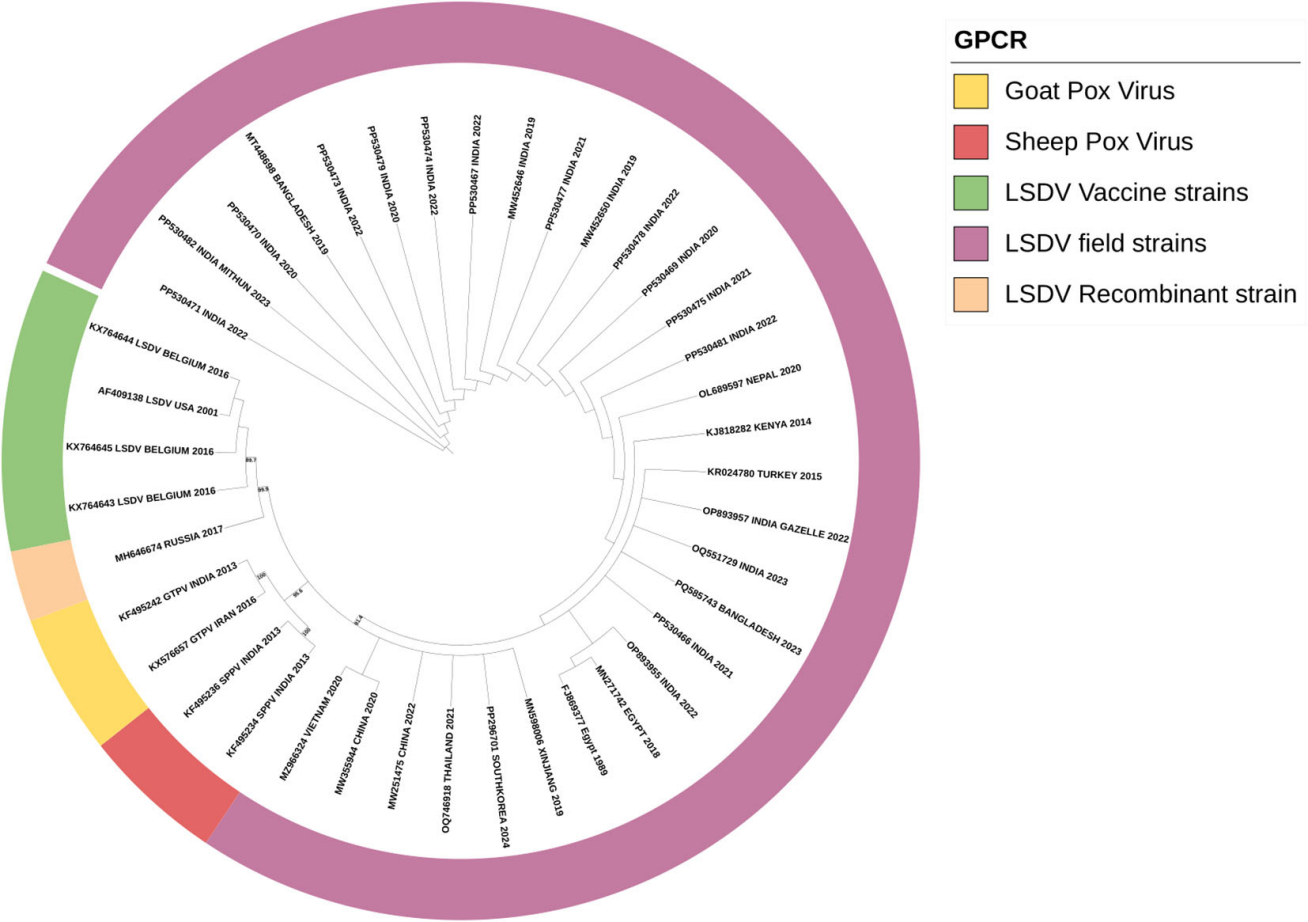
Phylogenetic analysis of the full-length gene of GPCR of the LSDV isolates obtained in this study suing maximum-likelihood tree showing the LSDV isolates close relationship with GPV than SPV.

DNA extracted from suspected cases and amplified for the GPCR gene produced a 1200 bp PCR product. Multiple sequence alignment of the GPCR gene of our isolates, reference sequence, and sheep and goat pox viruses has revealed the insertion of 21 bp in our isolates, confirming that our sequences are of LSDV. The resulting sequences were submitted to GenBank under accession numbers PP530466 to PP530481. The phylogenetic analysis of the GPCR gene has revealed that all the Indian sequences have clustered into a single clade, showing a common evolutionary origin. The sequences from the border countries of India, like Nepal and Bangladesh strains, form part of a broader clade indicating the entry of the virus from neighboring countries. The close genetic relationship between these South African strains points to the cross-border transmission of LSDV. The LSDV strain in Indian gazelle is particularly notable, the strain’s close relationship with the sequences of cattle-derived strains shows the inter-species transmission of LSD. The recombinant strains from Russia are tightly related to those from vaccine strains. This shows the rapidly evolving and transmission nature of LSDV. The recombinant strains, the sheep pox vaccine virus and goat pox vaccine virus formed separate clades as shown in **Figure 3**.

### 3.3 Whole genome assembly and annotation

*De novo* assembly with SPAdes using Illumina reads produced a total of 12 contigs. Reference-assisted contig assembly with Ragout yielded a single scaffold with a length of 150774 bp and 105 gaps. To fill the gaps, SOAPdenovo2-GapCloser was employed, resulting in a final genome of 150774 bp with a total of 20 gaps. Assembly coverage was calculated using Samtools depth by mapping the assembly against Illumina paired-end reads which resulted in an average depth of 1423.7. Per base coverage details are given in **Supplementary File Sheet 1**. The total coding region was 145986 bp with inverted terminal repeats of 2394 bp on either end, with an overall GC content of 25.9%. Annotation by GATU has predicted a total of 156 putative proteins.

### 3.4 Phylogenetic analysis

Whole genome sequences of LSDV strains were aligned with NCBI data to construct a maximum-likelihood phylogenetic tree and a maximum clade credibility tree, using goat pox virus and sheep pox virus as outgroups.

#### 3.4.1 Maximum likelihood analysis

The maximum likelihood tree shows the genetic diversity and evolutionary relationships between strains isolated from different geographical locations. The virus sequences formed multiple clades representing different geographical regions. This clustering suggests the region-specific evolution of the virus. Notably, strains from China and Vietnam form distinct sub-clades, indicating localized transmission and evolution. The strains from Vietnam share a close evolutionary relationship with the virus strains of Thailand and China. This shows the role of Southeast Asia in virus spread due to shared agricultural practices and livestock trade.

**Figure 4:**
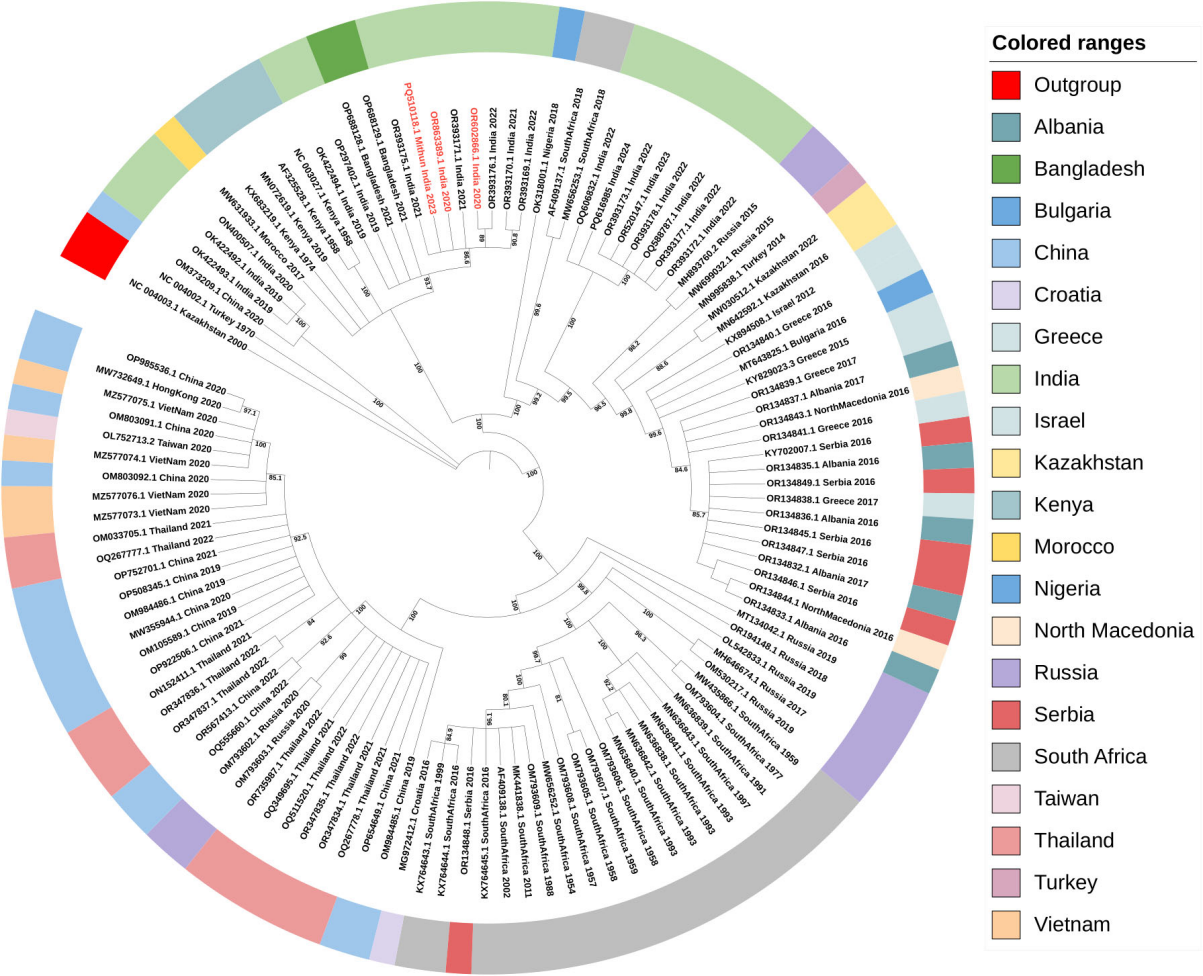
Phylogenetic analysis of the worldwide LSDV full genome sequences. The maximum likelihood tree was constructed using whole genome sequences. The LSD viruses from India (red color) are originated and evolutionarily more related to LSDV isolates from Kenya.

The LSDV strains from India are positioned on three distinct branches of the phylogenetic tree. Strains from the 2019 outbreaks cluster on one branch, closely related to the Neethling reference strain, with fewer genomic variations. The virus strain sequenced in this study (OR863389, PQ510118 and PQ616985) and previously sequenced in the lab (OR602866) also fall within this branch. In contrast, the LSDV strains from the recent 2022 outbreak form a separate branch and show a closer relationship to the Russian strains, displaying higher genomic variation, as illustrated in **Figure 4**. The results also demonstrated that the LSDV isolates from cattle to Mithun (*Bos Frontalis*) shared genomic similarity suggesting similar LSDV isolates are circulating in both cattle and Mithun.

#### 3.4.2 TMRCA estimation

The earliest TMRCA dates back to the early 17th century, as shown in the **Supplementary Figure 1**, reflecting a long evolutionary history of the virus. LSDV, initially endemic to Africa, has spread worldwide, particularly in regions such as Europe, Asia, and the Middle East.

The close clustering of strains from India and Kenya (NC_003027.1) suggest that Kenya has acted as a historical reservoir for LSDV, facilitating the virus’s spread to the Indian subcontinent, showing the global geographic Spread of LSDV. The viral strains sequenced in this study, OR863389, PQ510118, and OR602866, showed a TMRCA of early 2017. Multiple viral strains from India cluster together with a relatively recent TMRCA dating back to the last decade, showing that the virus is being introduced into the country through livestock movement.

#### 3.4.3 Phylogeny-associated variant landscape of LSDV genomes

Phylogenetic analysis revealed the presence of eight distinct clusters among the isolates. In total, 2116 single nucleotide polymorphisms (SNPs) and 106 insertions/deletions (indels) were detected across the genome. Notably, 294, 155, 15, 67 and 97 unique variants were identified in clusters 1.1, 1.2, 1.2_KSGPO, 2.1 and 2.5 respectively, indicating possible cluster-specific mutations contributing to the observed phylogenetic separation. Comprehensive details of these variants are provided in **Supplementary File** (**Sheet 2** and **Sheet 3**).

Cluster 1.1, comprising strains majorly from Africa, showed 294 unique variants, predominantly synonymous mutations, mainly located in genes LSDV144-156. Cluster 1.2 (Mediterranean like), with isolates from India, Russia and Mediterranean countries, had 155 unique variants, most of which were missence followed by synonymous mutations distributed across the genome. Cluster 1.2_KSGPO (KSGP-like) included isolates predominantly from India and Bangladesh, had 155 unique variants, most of which were missence mutations distributed across the genome. Cluster 2.1, had 67 variants mainly located in genes LSDV145– LSDV155, followed by LSDV001–008. Cluster 2.5, containing only Asian isolates, had 97 variants, mostly missense, with more than half observed in LSDV144–156. Indian isolates grouped into Clusters 1.2 and 1.2_KSGPO, with both of the isolates assembled in the study falling under the latter.

#### 3.4.4 Phylogenetic network analysis

**Figure 5:**
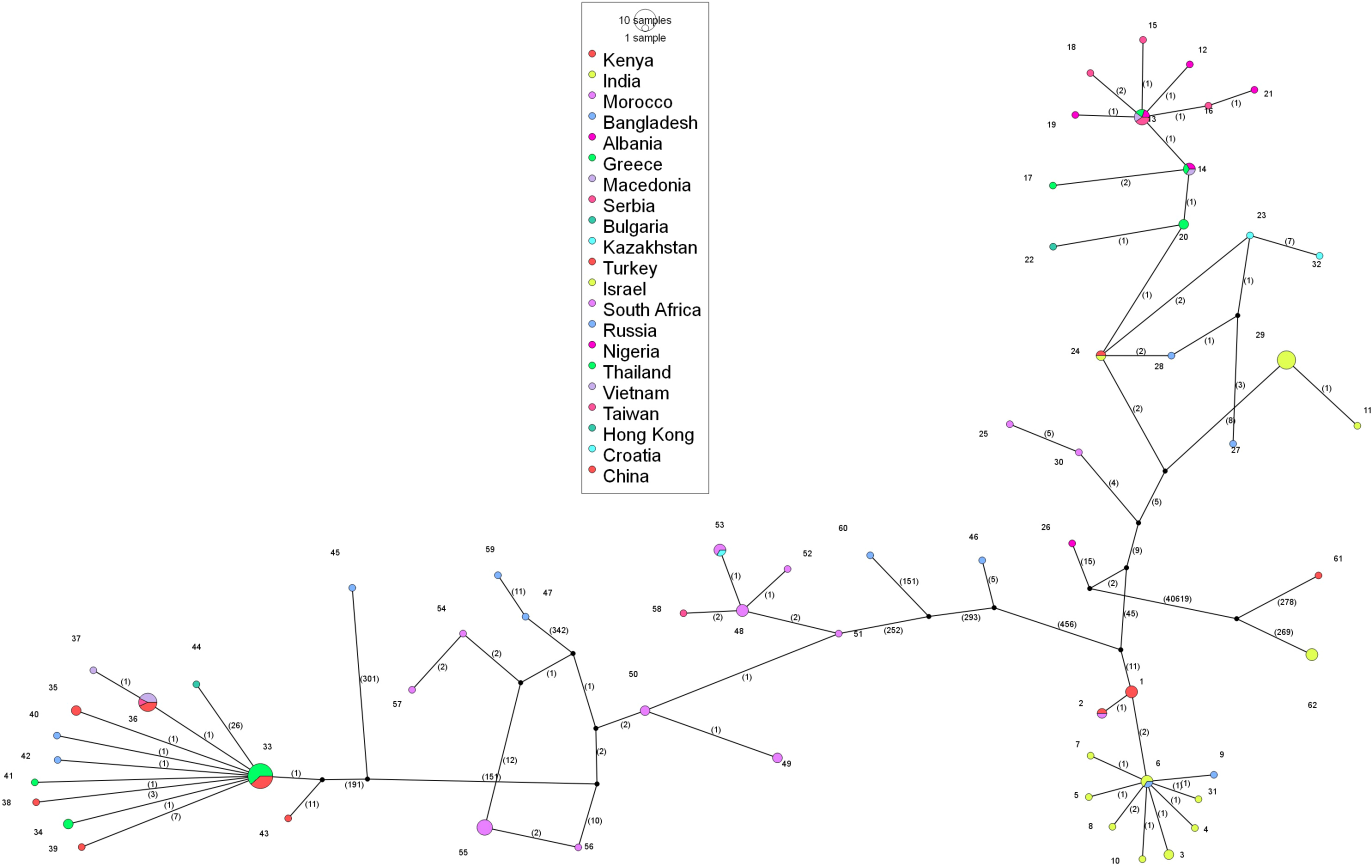
Analysis of phylogenomic geographic network of lumpy skin disease virus genomes 812 worldwide. Each of the circles represents either haplogroup or haplotype. The circle size states 813 the sequence load; the bigger the circle size represents the number of genome sequences there.

The multiple sequences alignment of the Lumpy skin disease virus and their subsequent analysis has revealed a total of 41594 segregating sites of which the PI sites were 41003. The incidence of sites and their distribution across gaps and ambiguous sequences, along with the statistical evaluation (Tajima’sD), has been summarized in **Table 1**. The Tajima’s D value of 0 indicates that the DNA sequence is evolving positively with a balancing evolution, meaning there is certain genetic variations being favored and maintained in the population. The constructed phylogeographic network is shown in the **Figure 5**.

**Table 1:** Tajima’s D statistical analysis of the variations in the studied genomes.

Sl. no	Network type	Number of segregating sites	Number of PI sites	Nucleotide diversity	Tajima’s D statistic
1	Transitive Consistence Score	41594	41003	0.0778324	D = -1.98337 p (D >= -1.98337) = 0.988641

The analysis of the phylogeographic network revealed that the predominant cluster is haplotype 33, with a total of 13 identical sequences from Thailand and China, followed by haplotype 36, with 7 identical sequences from China, Vietnam, and Taiwan. India has 11 different haplotypes circulating in the country with haplotype 29 containing 7 identical sequences. The viral strains isolated in the present study, OR863389, PQ510118, and OR602866, which are closer in the phylogenetic tree, belong to three different haplotypes, i.e., haplotype 6, 7, and 10, respectively. South Africa has a total of 12 haplotypes, Russia has a total of 9 haplotypes, and China has 7 haplotypes circulating in the country. Haplotypes 13 and 14 are present in the Balkan countries. Out of a total of 62 haplotypes, 43 haplotypes contain only a single sequence, and the rest are distributed in the remaining haplotypes with multiple sequences. Haplogroup-wise details of the identical sequences and their geographical locations are given in the **Supplementary File 1**.

## 4 Discussion

Lumpy skin disease is an important transboundary viral disease affecting cattle and buffalo with severe economic losses. It has been widespread in Africa, the Middle East, Europe, and Asia (Toplak et al., 2017; Mercier et al., 2018; Sprygin et al., 2020; Sudhakar et al., 2020) since its first report was made in Zambia, Africa in 1929 (Morris, 1930; MacDonald, 1931). Over the past decades, we have seen several vaccine spillovers, novel recombinant strains, and transboundary migration of LSDV. The high severity of LSDV in the recent outbreaks could be attributed to increased mutations in the LSDV genome, which has resulted in the circulation of multiple LSDV variants during the same time, as evident in the recent outbreaks from India (Yadav et al., 2024). The gaps in prior outbreaks hinder evolutionary inferences into the virus genome and constrain future vaccine development via harnessing routes of evolutionary machinery.

In this study, we have collected LSD outbreak samples from different states of India, namely, Karnataka, Maharashtra, Gujarat, Andhra Pradesh, and Madhya Pradesh, during the 2020–2022 outbreaks. The etiological agent was confirmed by the virus isolation as it is considered the gold standard test for LSDV identification (Amin et al., 2021), where the LSDV showed typical CPE of foci formation in the MDBK cell lines (Fay et al., 2020). Molecular detection was done by conventional PCR (Reddy et al., 2015; Sanjeevakumar et al., 2023), and preliminary phylogenetic analysis of the GPCR gene revealed that all the sequences were clustered into three main groups: LSDV, Sheep poxvirus, and Goat poxvirus cluster. Within the LSDV cluster, all the sequences in this study were clustered along with the field strains, standing distinctly from other LSDV recombinant and vaccine strains as reported in the previous reports (Sudhakar et al., 2020; Putty et al., 2023). This might be attributed to uncontrolled movement of cattle across different state province, mixed species rearing and grazing on natural pastures and also the mass vaccination with heterologous goatpox vaccine for prevention and control of LSD in India might have contributed for circulation of same field strain of LSDV, rather than vaccine and recombinant strains in India. Phylogenetic analysis of the specific genes, such as the GPCR gene, has been instrumental in constructing phylogenetic trees that depict the evolutionary lineage of LSDV and its relatives (Seerintra et al., 2022). These studies indicate that LSDV is more closely related to GTPV than to SPPV, suggesting a more recent common ancestry between LSDV and GTPV (Seerintra et al., 2022).

Vaccine strains, field isolates, and recombinant strains clustered distinctively and originated from a common ancestor, likely 400 years ago. This is in agreement with previous findings that the common ancestor of both LSDV clusters 1.1 and 1.2 existed ~ 500 years ago (Van Schalkwyk et al., 2022). The depth of this evolutionary history highlights the longstanding circulation of Capripoxviruses in animal populations, far preceding modern records of LSDV outbreaks. The phylogenetic results support theories that Capripoxviruses, including LSDV, share a common ancestor with GTPV and SPPV, which were also thought to have originated in Africa and possess unique genetic characteristics that contribute to their virulence and epidemiological behaviour (Ochwo et al., 2020; Ko et al., 2022). Early studies by Tulman et al. (2002) suggested that these viruses had been circulating in wild ungulate populations before their eventual spillover into domestic animals like cattle, goats, and sheep (Tulman et al., 2002). The ancient origin in the phylogenetic tree aligns with the proposition that sub-Saharan Africa served as a reservoir for various strains of Capripoxvirus. The virus’s persistence in this region likely facilitated its spread across Africa, the Middle East, and Europe over centuries (Babiuk et al., 2008a; Wang et al., 2022). This is further supported by the work of Gelaye et al. (2015), which highlighted the regional movement and adaptability of Capripoxviruses (Gelaye et al., 2015).

TMRCA implies that LSDV evolved over centuries of genetic adaptation before being identified as a prominent veterinary pathogen in the early twentieth century. Its evolutionary success is likely attributed to the virus’s genetic stability, which allowed it to persist in different environmental and ecological conditions. As a large DNA virus, LSDV has a slower mutation rate than RNA viruses, which helps maintain genomic integrity over time. Despite its slower mutation rate, LSDV shows ongoing genetic diversification, as evidenced by the different lineages in the phylogenetic tree. The emergence of newer strains in Russia, India, and China suggests that the virus continually adapts to new environments and hosts, possibly through recombination events with other field strains. This is supported by the findings of Kononova et al. (2021) and Suwankitwat et al. (2022), who demonstrated that recombination events can lead to more aggressive strains capable of causing severe outbreaks. The genetic diversity observed among LSDV strains is indicative of ongoing evolution, with studies highlighting the presence of mixed isolates that exhibit features of both vaccine and field strains (Chibssa et al., 2021; Suwankitwat et al., 2022). This genetic variability poses challenges for vaccine development and disease management strategies, as traditional vaccines may not provide adequate protection against newly emerged strains (Molla et al., 2017; Bedeković et al., 2018).

Similar estimations were provided for South African strains. The South African isolates from the 1950s, such as MW656252.1, have an estimated TMRCA from the late 1800s, which supports the theory that LSDV originated in sub-Saharan Africa. Some studies have traced the origins of LSDV to sub-Saharan Africa, with evidence of its emergence in various African countries before spreading to other regions (Bowden et al., 2008; Tuppurainen et al., 2017). The Indian sequences, particularly between 2019 and 2022 show a most common recent ancestor to be early 2017 and appear to have diverged from strains found in China and South Africa. This suggests India may have experienced multiple virus introductions, possibly through livestock trade routes. Tuppurainen et al. (2017) emphasize the role of the cattle trade in spreading LSDV from Africa to Asia. The Indian strain OR393172.1 and other strains in the cluster are closely related to strains from China (OM803091.1) and Russia (OM793603.1), suggesting recent viral spread across Asia, facilitated by livestock movement and transboundary trade. This aligns with the research of Ratyotha et al. (2022), who noted that LSDV outbreaks in South Asia were linked to cross-border livestock movement (Ratyotha et al., 2022). Although the Indian sequences shared a common ancestor, they still clustered into three different groups, depicting distinct evolutionary and recombination events. The first group comprised of two Ranchi isolates from 2019 and a Hyderabad isolate from 2020, accounting for the initial LSDV outbreaks in India. These isolates shared similar features with the then-Asian isolates. According to the previous reports, the second and third groups consisted of sequences majorly post-2021 and could be divided into low-mutation and highmutation isolates (Yadav et al., 2024). The second group clustered closer to the first, with the Neethling reference at the center, marking its evolution from the latter with lower mutations. This theory of early Indian isolates originating from Clade 1.2 Kenyan and derived KSGP strains from Eastern Africa has been confirmed by previous studies (Breman et al., 2023; Sudhakar et al., 2020). Our isolates clustered with the second group, affirming their origin from the early outbreaks. However, the situation has changed in the case of the third group, which seems to have evolved with the isolates from the 2015 Russian outbreak and showed high mutation rates compared to the reference strain.

A notable aspect of LSDV evolution is the emergence of recombinant strains, particularly in countries like Russia, China and Thailand (Ma et al., 2022; Seerintra et al., 2022). Recombinant viruses are significant because of their ability to adapt to new suitable hosts and can be more virulent. For example, Sprygin et al. (2018) reported similar recombinant strains in Russia, which likely contributed to the rapid spread and increased virulence of LSDV in the region (Sprygin et al., 2018). Shumilova et al. (2022) identified a similar recombinant strain in the Saratov region of Russia in 2019 that indicated the virus successfully overwintered the climatic conditions of Russia and caused the outbreak in the region, showing the constant survival and spread of the disease (Shumilova et al., 2022). In Thailand, recombinant strains such as OR347834.1 (Thailand 2021) suggest ongoing viral evolution, likely facilitated by the proximity of large livestock populations and frequent trade interactions between Thailand, India, and China. This is supported by Lubinga et al. (2014), who noted that the ability of LSDV to adapt rapidly through recombination has made its eradication in affected regions particularly challenging (Lubinga et al., 2014). Previous studies have highlighted the importance of recombination in the evolution of poxviruses like LSDV (Tuppurainen et al., 2017).

LSDV’s ability to spread and adapt across various regions is evident in its evolutionary trends. In China, the emergence of recombinant strains like OM803091.1 indicates the virus’s capacity to adapt to new regions and host populations. For instance, LSDV has been identified in several other species. Sudhakar et al. (2023) identified LSDV in freerange Indian gazelles, Reddy et al. (2023) identified LSDV in the yak, and more recently, LSDV was reported for the first time in Mithun by Reddy et al. (2024), this shows the ability of the poxviruses to undergo rapid genome recombination that might lead to their adaptation to new hosts as shown by Moss (1996), and particularly here in LSDV, the identification of the recombinant strain with combining sequences from a wild-type field strain and a vaccine strain in Russia by Sprygin et al. (2018) (Moss, 1996; Sprygin et al., 2018; Manjunatha Reddy et al., 2023; Sudhakar et al., 2023; Reddy et al., 2024). This identification of LSDV in different species suggests the possible interspecies transmission of LSDV just like other pox viruses, as seen in the case of PPRV (Lembo et al., 2013; Dao et al., 2022). This shows the host-jumping nature of the LSDV and further emphasizes LSDV’s adaptability to wide hosts.

The temporal aspect of LSDV evolution is also noteworthy, as the virus has displayed a pattern of increased incidence and geographical spread over the last few decades. For instance, significant outbreaks have been documented in the Middle East between 2012 and 2015, with a notable increase in cases reported in subsequent years (Alkhamis and VanderWaal, 2016). The emergence of LSDV in new regions, such as Southeast Asia and China, underscores the need for continuous monitoring and research to understand the dynamics of its evolution and spread (Wei et al., 2023).

DNA viruses are more stable and less frequently mutated than RNA viruses. However, the recent outbreaks of LSDV in the country are alarming and show the frequent mutation and recombination of the virus that happened in the previous decade. The number of mutations between these haplogroups demonstrates the virus’s inclination for genetic diversity. The cumulative variations in the genomes may have led to the recent epidemic observed in the country. Thus, continuous monitoring of the viral genome is necessary to control the disease. This study gives us an overview of the evolutionary network through haplogroups, haplotypes, and their geographical locations.

Phylogenetic analysis revealed the presence of eight distinct clusters among the isolates. The observed trends in the genetic variation across the clusters suggest several key inferences. First, the geographical distribution of isolates plays a significant role in shaping the variant landscape, with distinct patterns of genetic variation observed in different regions, such as India, China, Africa, and Eurasia. Synonymous mutations were predominant across most clusters, indicating a possible selective advantage in maintaining protein function while allowing for genetic diversity. The LSDV147–156 gene region emerged as a mutation hotspot, highlighting its potential importance in viral evolution and host adaptation. Notably, a recent pan-GWAS analysis further identified LSDV001/LSDV156, LSDV004/LSDV153 and LSDV002/LSDV155—as potential contributors to the presence or absence of pan-genome genes and observed phenotypes across different clades (Xie et al., 2024). Finally, the minimal genetic diversity observed in Cluster 1.2_KSGPO points to a conserved genomic structure or a recent common ancestor, suggesting limited variation within this group.

The TCS network analysis has revealed several distinct haplotypes are evident with clear geographic clustering. The reference sequence is grouped in Haplogroup 1, consisting of 3 genome sequences from Kenya. Haplotype 33 is the most prevalent group, with 13 sequences, followed by haplotype 29 and 36 with 7 identical sequences. South Africa has a maximum number of representations of haplotypes, with 12 from a total of 21 sequences, followed by India, with 11 from a total of 21 sequences. Haplotype 13 is present in 4 Balkan countries Albania, Greece, Serbia, and North Macedonia. The 3 Indian strains that formed a separate cluster in the phylogenetic tree are grouped in haplotype 62. This shows that multiple haplotypes are circulating in the regions. These results align with previous findings on capripoxviruses, where genetic diversity among strains is often shaped by livestock movement and trade (Tuppurainen and Oura, 2012). The geographical distribution of haplogroups provides interesting insights into LSDV distribution worldwide. The complex evolutionary dynamics found in LSDV, together with its genetic diversity, provide hurdles to effective disease control. While successful in some areas, current vaccinations may not significantly protect against all circulating strains. The geographical distribution of LSDV strains and the virus’s propensity to recombine and form new varieties underscores the need for regional, rather than worldwide, vaccine research efforts.

## 5 Conclusions

The phylogenetic analysis of the LSDV worldwide sequences provides a comprehensive understanding of LSDV’s evolutionary trajectory. From the study, it can be inferred that the virus has evolved through geographic diversification, recombination events, and host adaptation and the Indian strains have shown rapid diversification and share common ancestors with strains from not only Kenyan origin but also from China and Russia, reflecting cross-border transmission. The phylogenetic and haplotype network analysis also revealed species spillover of LSDV strains with circulation of geographically distinct haplogroups across India. The divergence between field and vaccine strains emphasizes the need for ongoing surveillance and vaccine updates to manage the spread of LSDV. The study highlights the importance of continued genetic surveillance in identifying emerging strains and informing vaccine strategies. The virus’s potential to recombine and adapt functionally emphasizes the need for region-specific management methods to prevent future outbreaks and reduce economic losses in affected countries.

## Data Availability

The datasets presented in this study can be found in online repositories. The names of the repository/repositories and accession number(s) can be found in the article/**Supplementary Material**.

## Supplementary material

Supplementary File 1 Haplogroup wise details of identical sequences in the study and their geographical distribution.

Supplementary File 2 Geographical distribution of the studied sequences and observed haplogroups.

Supplementary Figure 1 Estimated maximum-clade-credibility tree of LSD Virus. The Indian strains are marked in green, vaccine strains in blue, and outgroup in red colors.
