# Supplementary material for: Exploring the evolutionary journey of the lumpy skin disease virus through the phylogenetic and phylo-geo network analysis
Source: Front Cell Infect Microbiol. 2025 Jun 4;15:1575538. doi: 10.3389/fcimb.2025.1575538 (PMC12174415; doi:10.3389/fcimb.2025.1575538)
Supplement: Supplementary File 1 — Haplogroup wise details of identical sequences in the study and their geographical distribution. [file Table1.docx]

**Table 1:** Geographical distribution of the studied sequences and observed haplogroups.

| Sl.no | Country | Number of genomes | Sequences as haplogroups |
| --- | --- | --- | --- |
| 1 | Albania | 4 | 1 |
| 2 | Bangladesh | 2 | 1 |
| 3 | Bulgaria | 1 | 1 |
| 4 | China | 15 | 5 |
| 5 | Croatia | 1 | 1 |
| 6 | Greece | 5 | 2 |
| 7 | Hong Kong | 7 | 2 |
| 8 | India | 18 | 2 |
| 9 | Israel | 1 | 1 |
| 10 | Kazakhstan | 2 | 1 |
| 11 | Kenya | 3 | 1 |
| 12 | Morocco | 1 | 1 |
| 13 | Nigeria | 1 | 1 |
| 14 | North Macedonia | 1 | 1 |
| 15 | Russia | 4 | 3 |
| 16 | Serbia | 5 | 2 |
| 17 | South Africa | 18 | 2 |
| 18 | Taiwan | 1 | 1 |
| 19 | Thailand | 9 | 1 |
| 20 | Turkey | 1 | 1 |
| 21 | Vietnam | 4 | 1 |
|  | Total | 104 | 32 |

| Sl.no | Country | Number of genomes | Sequences as haplogroups |
| --- | --- | --- | --- |
| 1 | Albania | 4 | 1 |
| 2 | Bangladesh | 2 | 1 |
| 3 | Bulgaria | 1 | 1 |
| 4 | China | 15 | 5 |
| 5 | Croatia | 1 | 1 |
| 6 | Greece | 5 | 2 |
| 7 | Hong Kong | 7 | 2 |
| 8 | India | 18 | 2 |
| 9 | Israel | 1 | 1 |
| 10 | Kazakhstan | 2 | 1 |
| 11 | Kenya | 3 | 1 |
| 12 | Morocco | 1 | 1 |
| 13 | Nigeria | 1 | 1 |
| 14 | North Macedonia | 1 | 1 |
| 15 | Russia | 4 | 3 |
| 16 | Serbia | 5 | 2 |
| 17 | South Africa | 18 | 2 |
| 18 | Taiwan | 1 | 1 |
| 19 | Thailand | 9 | 1 |
| 20 | Turkey | 1 | 1 |
| 21 | Vietnam | 4 | 1 |
|  | Total | 104 | 32 |
