# Supplementary figures and images for: Exploring the evolutionary journey of the lumpy skin disease virus through the phylogenetic and phylo-geo network analysis
Source: Front Cell Infect Microbiol. 2025 Jun 4;15:1575538. doi: 10.3389/fcimb.2025.1575538 (PMC12174415; doi:10.3389/fcimb.2025.1575538)

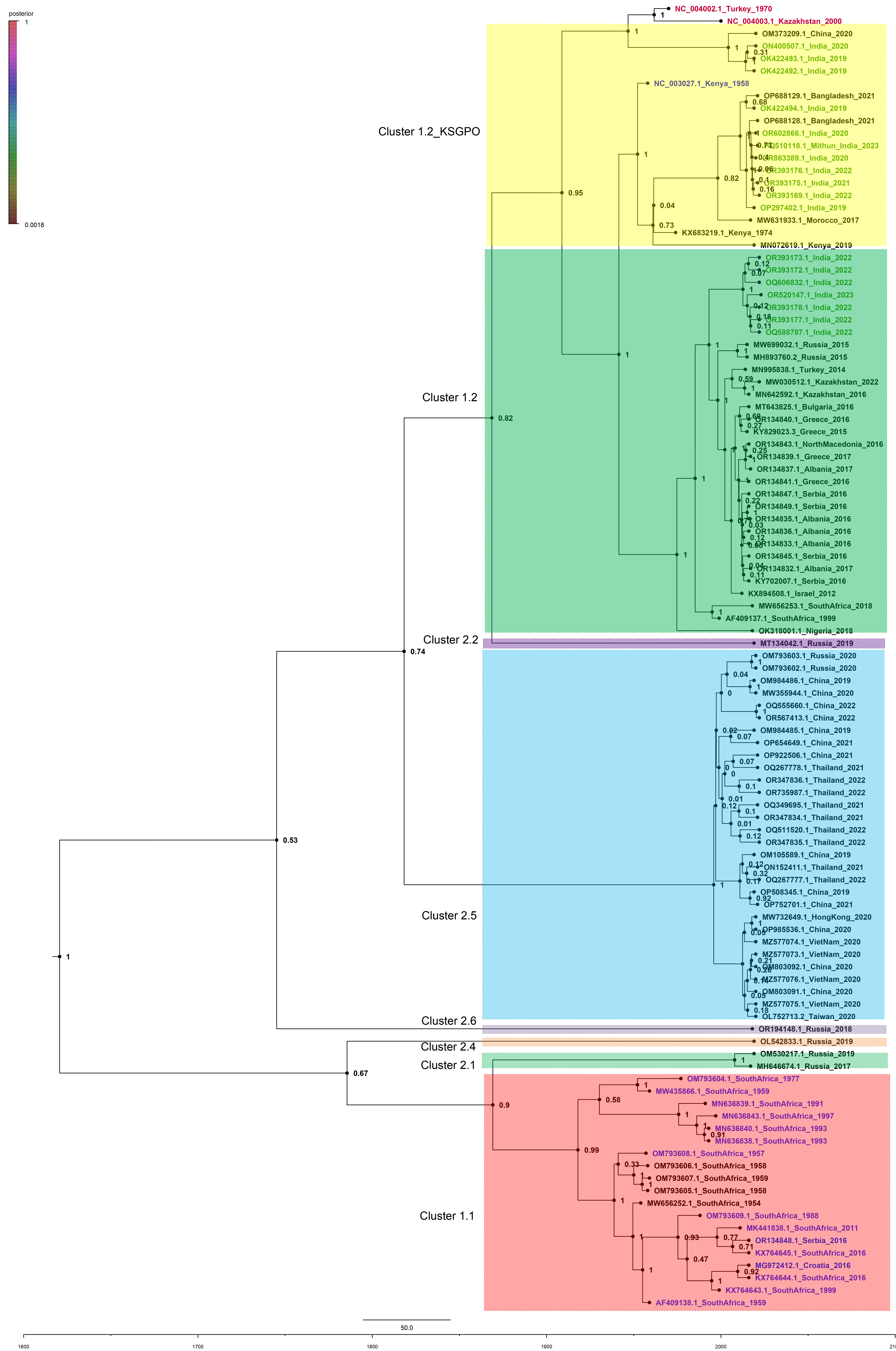

Supplement: Supplementary Figure 1 — Estimated maximum-clade-credibility tree of LSD Virus. The Indian strains are marked in green, vaccine strains in blue, and outgroup in red colors. [file Image1.jpeg]
